# Development of an Asymmetric Alginate Hydrogel Loaded with S-Nitrosoglutathione and Its Application in Chronic Wound Healing

**DOI:** 10.3390/gels11050354

**Published:** 2025-05-12

**Authors:** Jiafeng Tan, Minna Wen, Yifan Zhang, Shuyun Zhang, Min Fang, Junxiao Xiang, Xinshuo Liu, Jinhuan Tian, Lu Lu, Binghong Luo, Changren Zhou, Lihua Li

**Affiliations:** 1 Engineering Research Center of Artificial Organs and Materials, College of Chemistry and Materials, Jinan University, Guangzhou 510632, China; 15800015865@163.com (J.T.); 14778206314@163.com (M.W.); zhangyifan@jnu.edu.cn (Y.Z.); syj990842961303@163.com (M.F.); 17369242735@163.com (J.X.); lxs13393099365@163.com (X.L.); tlulu@jnu.edu.cn (L.L.); tluobh@jnu.edu.cn (B.L.); tcrz9@jnu.edu.cn (C.Z.); 2 College of Public Security and Traffic Management, Guangdong Police College, Guangzhou 510440, China; sunrain_zsy@163.com; 3 School of Life Sciences, Zhuhai College of Science and Technology, Zhuhai 519040, China

**Keywords:** S-nitrosoglutathione, asymmetrical hydrogel, sodium alginate, chronic wounds, infectious wounds

## Abstract

Nitric oxide (NO) is an endogenous signaling molecule that plays a critical role in wound healing. However, the gaseous nature, short half-life, and low stability of NO present challenges for its clinical application. To address these issues, this study introduces an innovative S-nitrosoglutathione (GSNO)-loaded asymmetric alginate (SA) hydrogel (GSNO-SA) as a novel solution for treating infected chronic wounds. The hydrogel is designed with a layer-by-layer melting-permeation crosslinking approach, forming a dense upper layer and a sparse lower layer structure, effectively promoting exudate management while delaying NO release. The results demonstrate that the GSNO-SA hydrogel extends NO release for up to 48 h, exhibits rapid exudate absorption (72.3 ± 1.5% equilibrium swelling after 5 min), significant antibacterial activity (over 90% antibacterial rate against *E. coli* and *S. aureus*), and anti-inflammatory effects (marked reduction in TNF-α expression), and promotes angiogenesis (90.00 ± 5.92% migration rate at 48 h). Additionally, animal studies show that the GSNO-SA hydrogel accelerates wound healing, achieving a 99.2 ± 0.1% closure rate at 14 days. Histological and immunohistochemical evaluations further confirm its ability to regulate inflammation (13.34-fold upregulation of CD163) and promote angiogenesis (3.02-fold upregulation of α-SMA). Theoretically, this asymmetric design provides a novel strategy for developing exudate-managing dressings by integrating controlled NO release with hierarchical pore structures.

## 1. Introduction

The treatment of chronic wounds, such as those caused by pressure ulcers, diabetic foot ulcers, and venous leg ulcers, has been a significant clinical challenge worldwide [1,2]. These wounds are typically associated with prolonged tissue ischemia and hypoxia, a high risk of bacterial infection, persistent inflammation, and excessive exudate, all of which severely hinder the healing process [3,4,5]. These factors present considerable challenges to conventional treatment approaches. Consequently, the development of a multifunctional dressing capable of absorbing exudate, exhibiting antibacterial and anti-inflammatory properties, and promoting angiogenesis is of paramount importance for improving the therapeutic outcomes of infected chronic wounds.

In recent years, nitric oxide (NO) has been increasingly applied in the treatment of chronic wounds [6]. NO is an endogenous signaling molecule that exhibits broad-spectrum antimicrobial properties at higher concentrations (μM), while at lower concentrations (nM to μM), it plays a role in modulating inflammation and promoting angiogenesis [6,7]. However, as a gaseous molecule, NO faces significant challenges in terms of transport, storage, and delivery. Its gaseous nature, short half-life, and low stability severely limit its clinical applicability [8]. To overcome these limitations, a range of NO donor molecules have been developed to optimize the direct application of NO gas, enhancing its stability and controllability [9]. The most widely employed NO donors include Nitrate esters of S-nitrosothiols (NONOates) and S-nitrosothiol (RSNOs) [10,11]. These two classes of donors are capable of spontaneously releasing NO under physiological conditions, which has led to their widespread use in the biomedical field [12]. Among them, NONOates exhibit poor chemical stability and are prone to decomposition during storage and use, which limits their broader application [9]. In contrast, RSNOs offer greater flexibility in controlling NO release. Their NO release is influenced by various factors, such as temperature and light, particularly ultraviolet and visible light, which effectively promote the homolytic cleavage of the S–N bond, thus releasing NO. This characteristic provides RSNOs with significant advantages in both NO release and storage [13]. S-nitrosoglutathione (GSNO), a type of RSNO, is naturally present in animal cells and serves as a natural NO donor with excellent biocompatibility. Compared to other RSNOs, GSNO functions more effectively in physiological environments, without causing significant immune rejection or toxic reactions. However, the release of NO from GSNO is too rapid under high temperature or light conditions, which does not align with the prolonged healing process of chronic wounds, thus presenting certain limitations in clinical applications [14,15,16]. Although numerous studies have been conducted on NO delivery systems, most of them exploited either its pro-angiogenic and anti-inflammatory effects at low concentrations [17,18,19] or its antimicrobial activity at high concentrations [20]. The multifunctional bioactivity of NO across different concentration levels has yet to be fully integrated and utilized.

**Figure 1:**
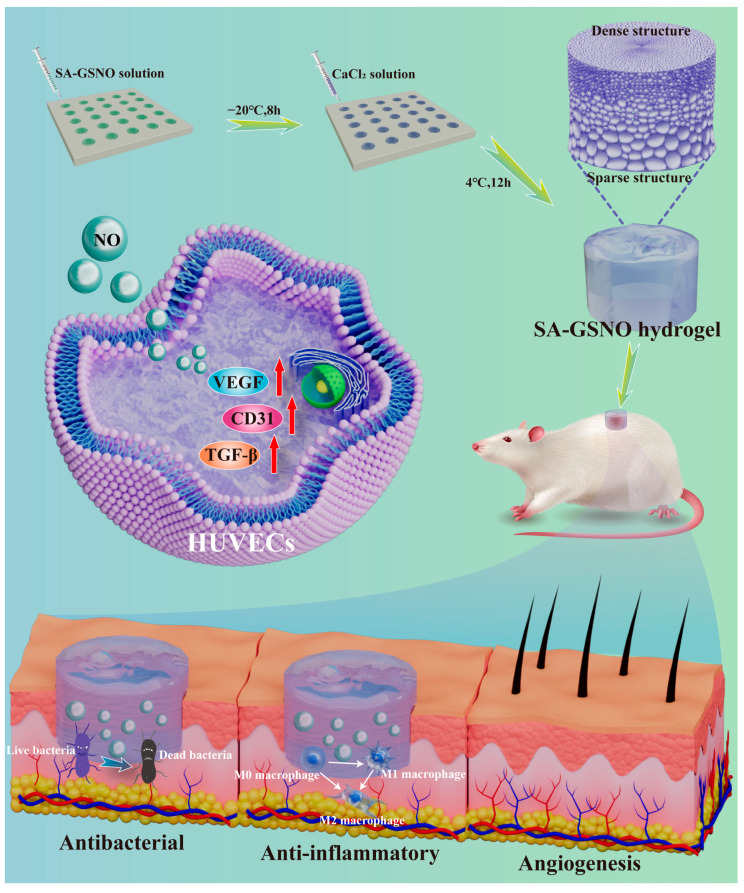
Schematic showing the preparation process and application of the GSNO-SA hydrogel.

To address the previously mentioned issues, this study proposed an innovative design of an asymmetric hydrogel loaded with GSNO (Figure 1). Sodium alginate (SA), a naturally derived polysaccharide with excellent biocompatibility, was rapidly crosslinked with Ca^2+^ within seconds [21]. A layer-by-layer melting-permeation crosslinking strategy was employed to fabricate a SA hydrogel with a dense upper layer and a sparse lower layer, forming an asymmetric structure. GSNO, which efficiently releases NO under physiological conditions, was incorporated into the hydrogel matrix, resulting in the GSNO-SA hydrogel. This design enabled controlled NO release while enhancing exudate management capabilities through the hydrogel’s asymmetric structure. By combining the antimicrobial, anti-inflammatory, and angiogenesis-promoting properties of GSNO with the exudate management functionality of the asymmetric SA hydrogel, this study provides a novel and efficient material platform. This platform holds potential for advancing clinical applications and improving treatment outcomes for patients with chronic wounds.

## 2. Result and Discussion

### 2.1. Characterization and Concentration Screening of GSNO

**Figure 2:**
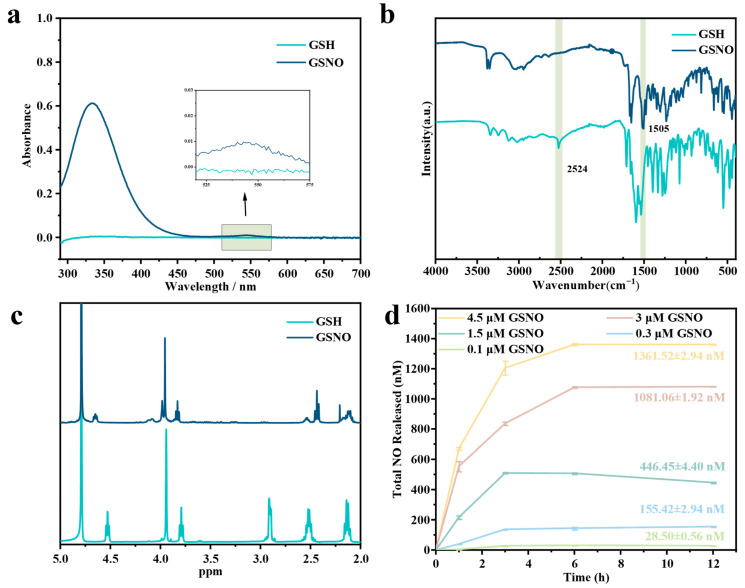
(**a**) UV–vis spectra of GSH and GSNO. (**b**) FTIR spectra of GSH and GSNO. (**c**) ^1^H NMR spectra of GSH and GSNO. (**d**) Cumulative release of GSNO.

Through spectral analysis, it was confirmed that GSNO had been successfully synthesized. In the UV–vis spectra (Figure 2a), absorption peaks at 337 nm (n_0_ → π* transition) and 545 nm (n_N_ → π* transition) are commonly used to determine the synthesis of GSNO [22]. In addition, the Fourier-transform infrared (FTIR) spectra of GSH and GSNO (Figure 2b) show distinct features. GSH exhibits a prominent absorption peak at 2524 cm^−1^ (S-H), while GSNO does not show a significant absorption peak in this region, but it shows enhanced absorption at 1505 cm^−1^ (N=O). Figure 2c displays the proton nuclear magnetic resonance (^1^H NMR) spectra of GSH and GSNO. At 2.95 ppm, GSH shows a peak corresponding to the methylene protons attached to the thiol group. After the reaction, due to the electron-withdrawing nature of the nitroso group, the peak shifts to a lower field, with the peak moving to 4.11 ppm.

Upon exposure to light and heat, the S-N bond in GSNO was cleaved, resulting in the rapid release of NO. The concentration of released NO was calculated using the standard curve (A = 0.00106C + 0.03872, R^2^ = 0.9999) (Figure S1). As shown in Figure 2d, within 0 to 6 h, a higher GSNO concentration resulted in a faster rate of NO release. The high-concentration groups (4.5 and 3.0 μM) underwent complete decomposition within 6 h, releasing 1361.52 ± 2.94 nM and 1081.06 ± 1.92 nM of NO, respectively. In contrast, the low-concentration groups (1.5, 0.3 and 0.1 μM) decomposed completely within 3 h, releasing 446.45 ± 4.40 nM, 155.42 ± 2.94 nM, and 28.50 ± 0.56 nM of NO, respectively. The cumulative NO release was found to be dependent on the GSNO concentration.

**Figure 3:**
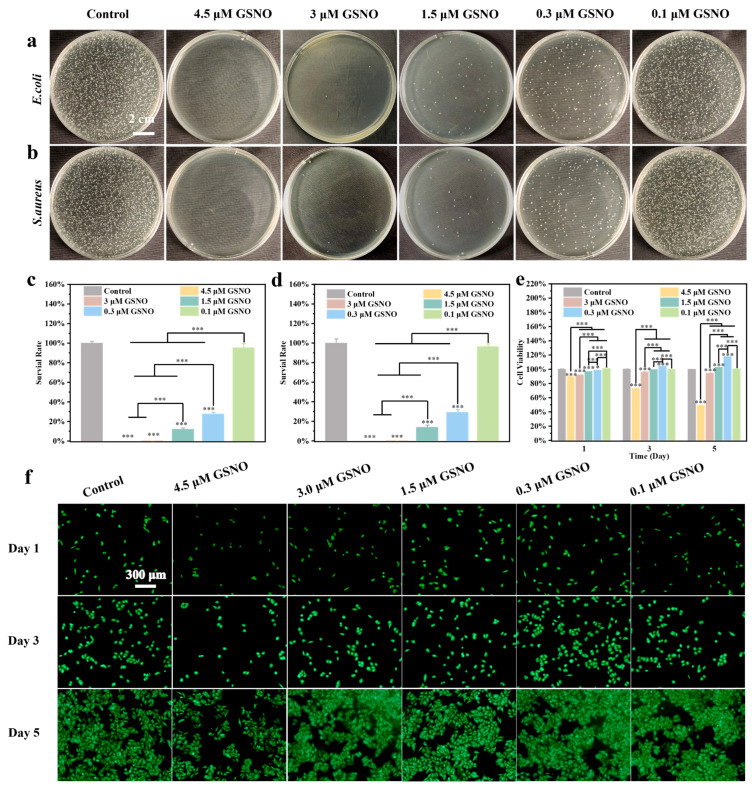
(Bacterial plates of GSNO at different concentrations ((**a**) *E. coli*, (**b**) *S. aureus*)). (Quantitative statistics of bacterial plates ((**c**) *E. coli*, (**d**) *S. aureus*)). (**e**) Cell viability of HUVECs treated with different concentrations of GSNO. (**f**) Live/dead staining images of HUVECs cells treated with different concentrations of GSNO. (*** *p* < 0.001, *n* = 3).

The effects of nitric oxide (NO) on wound healing vary with its concentration. At higher concentrations (μM), NO demonstrates broad-spectrum antimicrobial properties. In contrast, at lower concentrations (nM–μM), NO plays a key role in regulating various cellular behaviors involved in the wound healing process. Therefore, an appropriate concentration range for GSNO needed to be determined to fully exploit the functional potential of NO. *Escherichia coli* (*E. coli*) and *Staphylococcus aureus* (*S. aureus*), common pathogens responsible for wound infections, were utilized to evaluate the antimicrobial properties of GSNO by co-culturing various concentrations of GSNO with the bacteria. As shown in Figure 3a–d, 0.1 μM GSNO exhibited negligible antibacterial activity against *E. coli* and *S. aureus*. As the concentration of GSNO increased, its antibacterial activity progressively intensified. At a concentration of 0.3 μM, the survival rates of *E. coli* and *S. aureus* were both reduced to below 30%. At 1.5 μM, bacterial colonies were sparsely distributed on the agar plate, demonstrating a pronounced inhibitory effect. When the concentration exceeded 3.0 μM, it achieved nearly 100% antibacterial efficacy. The results indicated that the antibacterial activity of GSNO was positively correlated with its concentration; higher concentrations resulted in more pronounced antibacterial effects.

HUVEC cells were used to evaluate the cytocompatibility of GSNO. The live/dead staining images of HUVEC cells (Figure 3f) revealed that, on day 3, the cell density in the 4.5 μM GSNO group was significantly lower than in the other groups, indicating cytotoxicity. Additionally, the results of the CCK-8 assay for GSNO on HUVEC cells (Figure 3e) demonstrated that, on day 5, the cell survival rates in the 4.5, 3.0, 1.5, 0.3, and 0.1 μM GSNO groups were 49.3 ± 0.4%, 94.5 ± 0.6%, 103.5 ± 0.2%, 117.6 ± 0.2%, and 101 ± 0.6%, respectively. Except for the 4.5 μM GSNO group, which exhibited clear cytotoxicity, the survival rates in the other groups were all higher than 95%. Moreover, the cell viability of HUVECs exhibited a trend of initially increasing and then decreasing with the increasing concentration of GSNO. This suggests that the cell compatibility of the hydrogel is closely related to the GSNO concentration, with higher concentrations potentially inducing cytotoxic effects.

### 2.2. Characterization of GSNO-SA Hydrogel

**Figure 4:**
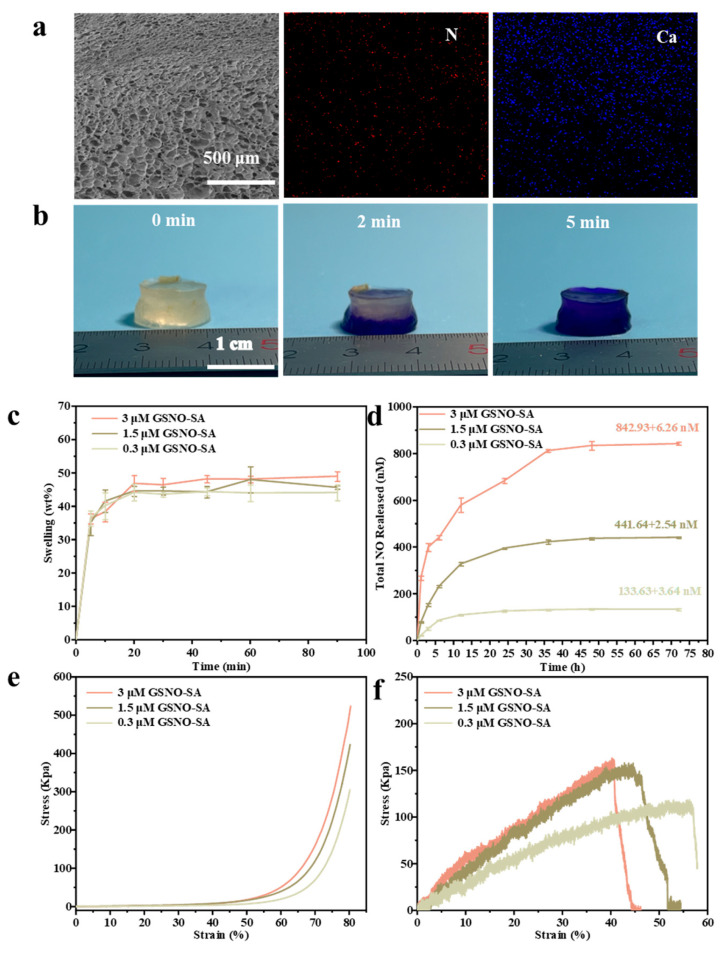
Characterization of the GSNO-SA hydrogel. (**a**) Mapping images of the GSNO-SA hydrogel. (**b**) Images of the GSNO-SA hydrogel absorbing exudate (the solution used in the images is PBS containing crystal violet). (**c**) Swelling ratio curve of the GSNO-SA hydrogel. (**d**) Cumulative release curve of the GSNO-SA hydrogel. (**e**) Compression stress–strain curve of the GSNO-SA hydrogel. (**f**) Tensile stress–strain curve of the GSNO-SA hydrogel.

Excessive tissue exudate was found to not only increase the risk of infection but also potentially induce an inflammatory response, thereby impairing the healing process. To address this challenge, a porous structure with a dense upper layer and a loose lower layer was designed to facilitate rapid exudate absorption. As shown in Figure 4a, the GSNO-SA hydrogel features a network of pores, with pore size increasing progressively from top to bottom. Furthermore, the nitrogen (N) distribution within the hydrogel is uniform, while the calcium (Ca) concentration decreases from the top to the bottom, consistent with the variation in pore size. The asymmetric structure of the GSNO-SA hydrogel is attributed to the synergistic mechanism of layered defrosting and crosslinking. The SA solution rapidly crosslinked with Ca^2+^ [21]; therefore, pre-freezing the hydrogel precursor solution was employed to slow down the crosslinking process. When a CaCl_2_ solution at room temperature was dropped onto the surface of the frozen precursor solution, the top layer thawed first and formed a dense crosslinked network upon contact with the high concentration of Ca^2+^. In contrast, the deeper layers, due to their lower temperature, thawed gradually with the increasing temperature gradient, leading to a reduced Ca^2+^ diffusion flux and, consequently, a decrease in crosslinking density.

Figure 4b visually illustrates the superior and rapid exudate absorption capacity of the GSNO-SA hydrogel. At 2 min, the bottom of the hydrogel had already turned completely purple due to the uptake of crystal violet-containing PBS, and by 5 min, the entire hydrogel exhibited a uniform purple coloration. These results indicate that exudate was rapidly absorbed and effectively retained by the hydrogel. Figure 4c presents the swelling behavior of the hydrogel during exudate absorption. The swelling ratio increased sharply within the initial minutes, reaching 72.3 ± 1.5% of the equilibrium value at 5 min. Near-complete equilibrium was achieved by 20 min, and the swelling behavior stabilized within 60 min. These findings confirmed the hydrogel’s ability to rapidly absorb and continuously store exudate. This performance was attributed to the asymmetric porous structure of the GSNO-SA hydrogel, characterized by a densely packed upper layer and a more porous lower layer. The small, densely distributed pores in the upper layer enhanced capillary action, enabling rapid fluid uptake and accelerating the initial absorption rate. Additionally, the microporous network minimized fluid loss and improved overall absorption efficiency. In contrast, the larger and more sparsely distributed pores in the lower layer facilitated rapid diffusion and provided storage capacity, reducing resistance to fluid uptake, preventing local saturation, and ensuring uniform exudate distribution throughout the matrix.

To evaluate the in vitro release behavior of GSNO-SA hydrogel at 37 °C, both cumulative and real-time release experiments were performed. As shown in Figure 4d, the dense three-dimensional network of the SA hydrogel significantly delayed nitric oxide (NO) release through a physical confinement effect, in contrast to the rapid decomposition of free GSNO, which was completely released within 6 h. The release duration was extended to 48 h for the hydrogel. The 3.0 μM group exhibited a typical biphasic release profile, characterized by a rapid release phase within the first 12 h, followed by a sustained release plateau. Notably, the release rate during the plateau phase (23.08 ± 5.02 nM/h) closely mirrored the initial burst release rate observed in the 0.3 μM group (22.09 ± 1.47 nM/h) (Figure S2 and Table S1, Supporting Information). This release behavior was attributed to the high GSNO concentration, which enhanced the crosslinking density of the hydrogel, suppressed swelling, and slowed the dissociation of Ca^2+^-SA coordination bonds. These factors collectively reduced the degradation of GSNO and the diffusion of NO, leading to prolonged and controlled release. To better evaluate the release kinetics of NO, the cumulative release profiles were fitted using the Korsmeyer–Peppas model, as shown in Figure S4. The correlation coefficients (R^2^) for the respective groups were 0.9819, 0.9304, and 0.8990, and the diffusion exponent (n) values were all below 0.5, indicating that the release of NO was governed by Fickian diffusion in accordance with the Korsmeyer–Peppas model [23].

Hydrogels must exhibit sufficient mechanical properties to prevent damage during use. In terms of compressive performance, Figure 4e demonstrates that hydrogels with higher GSNO concentrations can withstand greater compressive stress. The compressive strength of the 3 μM GSNO-SA hydrogel was 466.36 ± 33.24 kPa, whereas the 0.3 μM GSNO-SA hydrogel showed a lower compressive strength of 262.35 ± 35.56 kPa (Figure S3a). Regarding tensile properties (Figure 4f), the 3 μM GSNO-SA hydrogel maintained higher tensile stress even at larger strains, while the 0.3 μM GSNO-SA hydrogel fractured at lower strains, indicating poor toughness. The tensile strength of the 3 μM GSNO-SA hydrogel was 200.54 ± 25.04 kPa, whereas the 0.3 μM GSNO-SA hydrogel exhibited a tensile strength of 70.12 ± 34.41 kPa (Figure S3b). The enhanced mechanical stability of the GSNO-incorporated hydrogels was attributed to strengthened intermolecular interactions, such as hydrogen bonding and electrostatic forces.

### 2.3. In Vitro Antibacterial Activity of GSNO-SA Hydrogel

**Figure 5:**
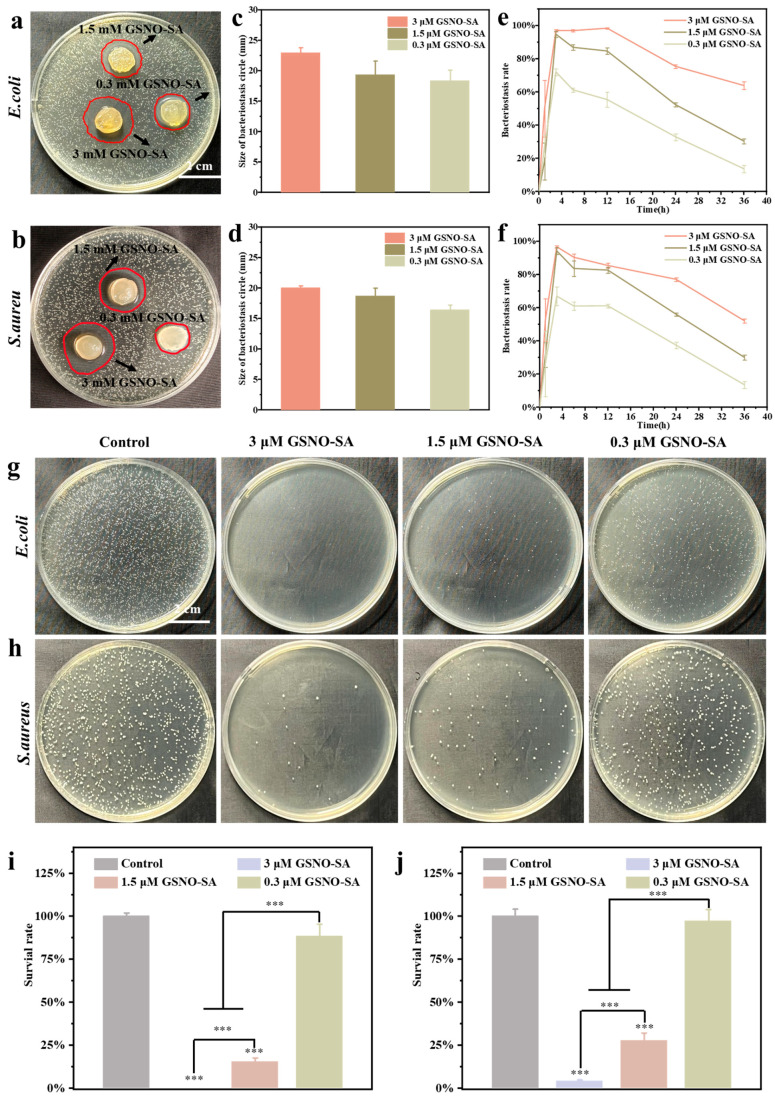
(Inhibition zone assay results of the GSNO-SA hydrogel ((**a**) *E. coli*, (**b**) *S. aureus*)). (Statistical analysis of inhibition zone diameters ((**c**) *E. coli*, (**d**) *S. aureus*)). (Bacterial plates after treatment with the GSNO-SA hydrogel ((**e**) *E. coli*, (**f**) *S. aureus*)). (Bacterial plates of GSNO at different concentrations ((**g**) *E. coli*, (**h**) *S. aureus*)). (Quantitative statistics of bacterial plates ((**i**) *E. coli*, (**j**) *S. aureus*)). (*** *p* < 0.001, *n* = 3).

To evaluate the antibacterial performance of the GSNO-SA hydrogel, inhibition zone assays, bacterial growth curves, and plate spreading methods were employed to assess its inhibitory effects against *E. coli* and *S. aureus*. The results indicated that the GSNO-SA hydrogel exhibited significant antibacterial activity against both bacterial strains, with enhanced efficacy observed at higher GSNO concentrations. As shown in Figure 5a,b, the inhibition zone of the 3 μM GSNO-SA hydrogel group was markedly larger than those of the other groups, suggesting that higher GSNO concentrations released greater amounts of NO, thereby more effectively suppressing bacterial growth. Figure 5c,d further confirms that the 3 μM group yielded the largest inhibition zones, whereas the 0.3 μM group demonstrated only weak antibacterial effects.

In the antibacterial curve assay (Figure 5e,f), the 3 μM GSNO-SA hydrogel exhibited inhibition rates of 98.3 ± 0.3% against *E. coli* and 82.3 ± 1.5% against *S. aureus* within 12 h, whereas the 0.3 μM group showed significantly lower inhibition rates of only 54.2 ± 6.7% and 59.6 ± 0.7%, respectively. Although the antibacterial efficacy of all groups gradually declined over time, the 3 μM hydrogel maintained high inhibition levels for an extended duration, with the 1.5 μM group retaining an inhibition rate above 80% within 12 h, while the efficacy of the 0.3 μM group decreased rapidly. Plate spreading results (Figure 5g–j) showed dense bacterial colonies in the control group, whereas the 3 μM GSNO-SA hydrogel group exhibited sparse colonies (The survival rates of *E. coli* and *S. aureus* were reduced to nearly 0% and 4.2 ± 0.8%, respectively), indicating a pronounced antibacterial effect. The 1.5 μM group showed a moderate reduction in colony formation (the survival rates of *E. coli* and *S. aureus* were reduced to 15.3 ± 2.5% and 27.6 ± 4.6%, respectively), while the 0.3 μM group displayed relatively weak antibacterial performance.

### 2.4. Cell Biocompatibility and In Vitro Cell Migration of GSNO-SA Hydrogel

**Figure 6:**
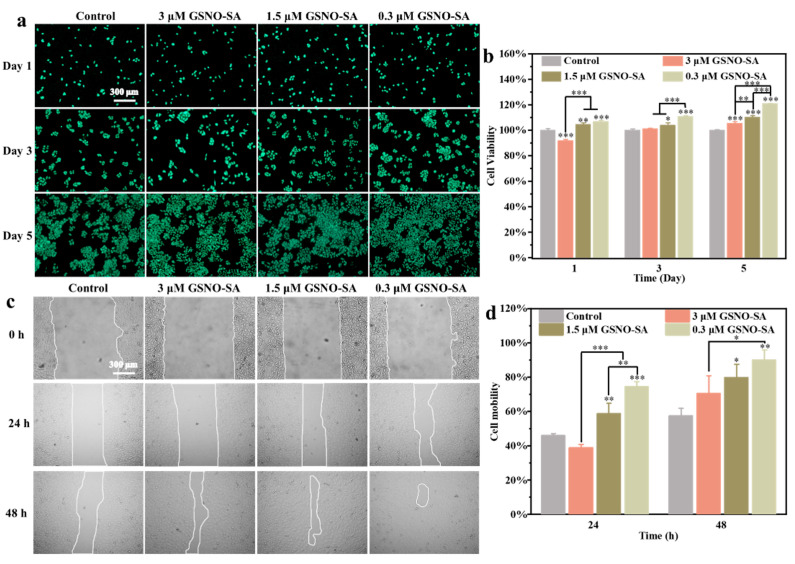
(**a**) Live/dead staining of HUVECs cells after treatment with the GSNO-SA hydrogel. (**b**) Viability of HUVEC cells after treatment with the GSNO-SA hydrogel. (**c**) Schematic of cell scratch assay for HUVECs after the GSNO-SA hydrogel treatment. (**d**) Cell migration rate. (* *p* < 0.05, ** *p* < 0.01, *** *p* < 0.001, *n* = 3).

The cytocompatibility of the hydrogel systems was assessed through CCK-8 assays and AO staining. As shown in Figure 6a,b, in the early cultivation phase (Day 1), the cell survival rate of HUVECs in the 3 μM group was 12% lower than that in the control group, likely due to transient oxidative stress induced by the burst release of high-concentration NO (>250 nM). However, by Day 5, the survival rate in this group exceeded that of the control group. The low-concentration groups (1.5, 0.3 μM) displayed a progressive proliferative pattern, particularly the 0.3 μM group, which showed a 30% increase in cell density compared to the control group at Day 5. This behavior closely corresponded to the NO release kinetics, which indicated that early high-concentration NO alleviated the inflammatory microenvironment by inhibiting the NF-κB pathway, while later low-dose NO (<100 nM) promoted angiogenesis via activation of the VEGF/VEGFR2 signaling axis [24]. Additionally, the hydrogel was co-cultured with Hacat and L929 cells, as shown in Figures S5 and S6. Over a 5-day period, all experimental groups exhibited excellent cell compatibility, with cell viability consistently remaining above 90%.

Endothelial cell migration is critical for wound healing, as these cells regulate vascular homeostasis through autocrine and paracrine signaling. Figure 6c,d shows that, after 48 h of incubation, the migration rates in the 0.3, 1.5, and 3 μM groups were 70.40 ± 10.55%, 79.77 ± 7.87%, and 90.00 ± 5.92%, respectively, representing a 1.2- to 1.6-fold increase compared to the control group (57.35 ± 4.63%) (*** *p* < 0.001). Notably, at 24 h, the migration rate in the 3 μM group was 35%, significantly lower than that of the control group (50%) (*** *p* < 0.001). This phenomenon is closely linked to the NO release kinetics (Figure 4d); the initial burst of high-concentration NO (>300 nM) transiently delays cell lamellipodia formation by inhibiting the RhoA/ROCK pathway, while the later maintenance of NO at physiological levels (~80 nM) activates the VEGFR2/FAK signaling axis to promote directional migration [25].

### 2.5. PCR Analysis of Inflammatory Factors and Angiogenesis-Related Factors

**Figure 7:**
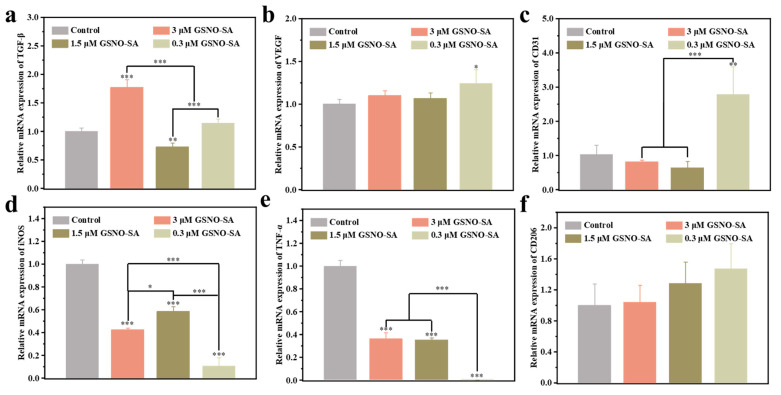
Expression of (**a**) TGF-β, (**b**) VEGF, and (**c**) CD31 genes in HUVECs after the GSNO-SA hydrogel treatment. Expression of (**d**) iNOS, (**e**) TNF-α, and (**f**) CD206 genes in RAW264.7 cells after the GSNO-SA hydrogel treatment. (* *p* < 0.05, ** *p* < 0.01, *** *p* < 0.001, *n* = 3).

The expression of cellular angiogenesis-related and inflammation-related factors was detected using cellular expression polymerase chain reaction (PCR). The pro-angiogenic potential of the hydrogel was first evaluated using PCR (Figure 7a–c). VEGF, CD31, and TGF-β are well-established pro-angiogenic factors [26,27,28]. The expression of VEGF and CD31 was significantly higher in the 0.3 μM GSNO-SA hydrogel group compared to the other groups (* *p* < 0.05 or *** *p* < 0.001), suggesting that this concentration effectively promoted angiogenesis. Additionally, the 3 μM GSNO-SA hydrogel group predominantly upregulated TGF-β (***p < 0.001), indicating that this concentration also facilitated vascular formation. In contrast, the 1.5 μM GSNO-SA hydrogel group exhibited lower expressions of VEGF and CD31, with no significant upregulation of TGF-β, demonstrating a relatively weaker effect.

Then, to comprehensively evaluate the effects of the hydrogel on inflammation cells RAW264.7, the expression of pro-inflammatory factor TNF-α, iNOS, and anti-inflammatory factor CD206 were investigated (Figure 7d–f) [29,30,31]. In all GSNO-SA groups, TNF-α expression was significantly inhibited (*** *p* < 0.001), indicating that the GSNO-SA hydrogel, at various concentrations, exhibited potent anti-inflammatory activity. The expression of iNOS was also significantly reduced across all groups (*** *p* < 0.001), suggesting that the reduction in iNOS may reflect the hydrogel’s role in mitigating excessive NO production, thereby preventing an exaggerated inflammatory response and contributing to its anti-inflammatory effects. No significant differences in CD206 expression were observed among the groups, although the GSNO-SA hydrogel groups showed higher levels compared to the control group. This suggests that the hydrogel may promote the polarization of M2 macrophages, potentially aiding in wound healing.

### 2.6. Assessment of In Vivo Wound Healing and Histological Evaluation

**Figure 8:**
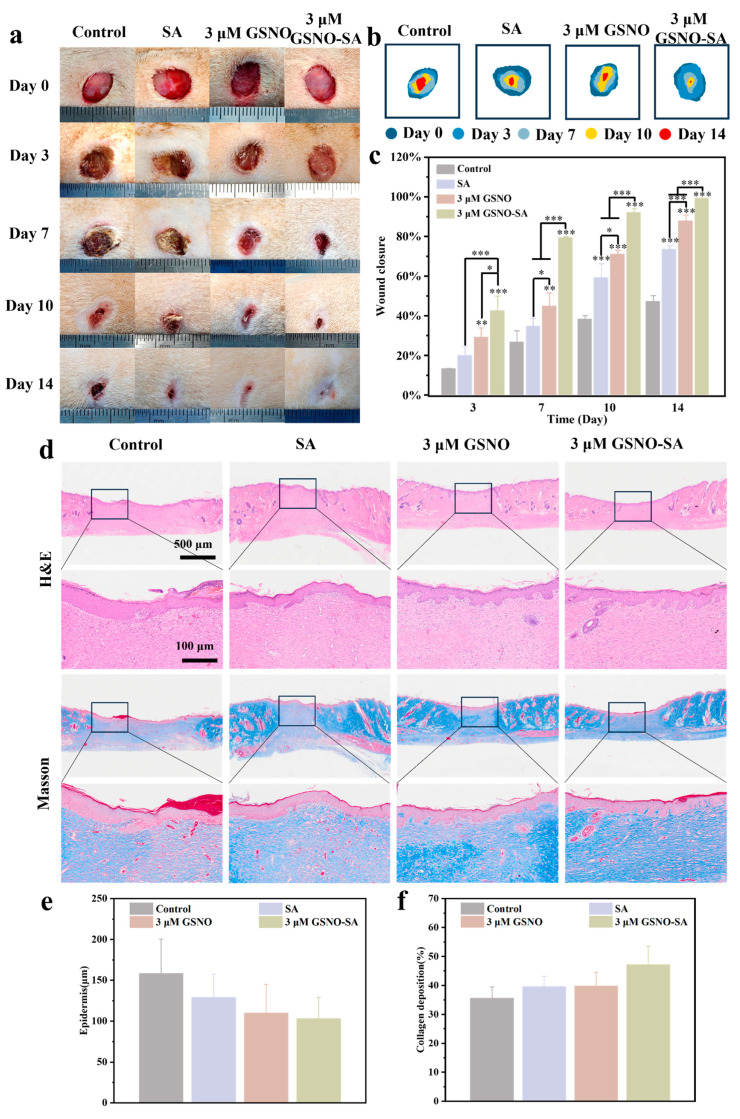
(**a**) Wound photographs at different time points. (**b**) Schematic diagram of wound healing. (**c**) Wound healing rate. (**d**) H&E staining and Masson staining images of wounds in different groups on day 14. (**e**) Quantitative analysis of epidermal thickness in different groups. (**f**) Quantitative analysis of collagen deposition in different groups. (* *p* < 0.05, ** *p* < 0.01, *** *p* < 0.001, *n* = 3).

An infectious pressure ulcer model was established to assess the potential of the 3 μM GSNO-SA hydrogel in the treatment of chronic wounds. As shown in Figure 8a,b, the control group exhibited the slowest wound healing, with severe inflammation, likely due to persistent infection impairing tissue repair. The SA group showed some improvement in wound healing, indicating that the hydrogel’s exudate management helped optimize the wound environment. However, its lack of direct antibacterial activity limited its healing effect. In contrast, the 3 μM GSNO group demonstrated significantly faster healing, likely due to the antibacterial, anti-inflammatory, and angiogenic effects of GSNO through NO release, which promoted cell migration and tissue repair. The 3 μM GSNO-SA group displayed substantial healing at day 10 and nearly completed healing by day 14, showing the most effective therapeutic outcome. The statistical analysis, presented in Figure 8c, revealed that the control group had the lowest wound closure rate. The SA group showed slight improvement, but the difference was not significant, suggesting that while the hydrogel improved the wound environment, it did not accelerate healing. In contrast, the 3 μM GSNO group demonstrated a significantly higher healing rate at both days 7 and 10 compared to the control and SA groups. The 3 μM GSNO-SA group achieved the highest closure rate throughout the experiment, with a healing rate of 99.2 ± 0.1% at day 14, compared to only 43.2 ± 3.2% in the control group.

The wound healing process was further evaluated through H&E staining, Masson staining, and the quantitative analysis of tissue samples from the wound site at day 14. As shown in Figure 8d–f, H&E staining indicated that, by day 14, reepithelialization had been completed in all groups. However, the epidermis in the control group was the thickest, measuring 158.67 ± 41.74 μm, while the epidermis in the SA group and the 3 μM GSNO group was thinner. The thinnest epidermis was observed in the GSNO-SA group, with a thickness of 103.33 ± 26.03 μm, representing a 34.88% reduction compared to the control group. This could be attributed to the persistent inflammatory response in the control group, which induced epidermal hyperplasia [32]. In contrast, the GSNO-SA hydrogel treatment appeared to regulate inflammation and promote tissue repair, leading to an epidermis with a closer-to-normal thickness and suggesting superior healing quality. Masson staining showed that, by day 14, collagen deposition in the control group was still limited, and the tissue structure had not fully recovered, indicating a slow healing process. The SA group demonstrated increased collagen deposition, but the fiber arrangement remained loose. In the 3 μM GSNO group, collagen deposition was almost mature, and the tissue structure was becoming more stable. The 3 μM GSNO-SA group exhibited the most uniform blue areas, with widespread collagen distribution. Collagen deposition density in this group was 1.33 times higher than in the control group, indicating the formation of a more complete collagen fiber network in the final stage of wound repair, thereby promoting full wound healing.

### 2.7. In Vivo Antibacterial Effect

**Figure 9:**
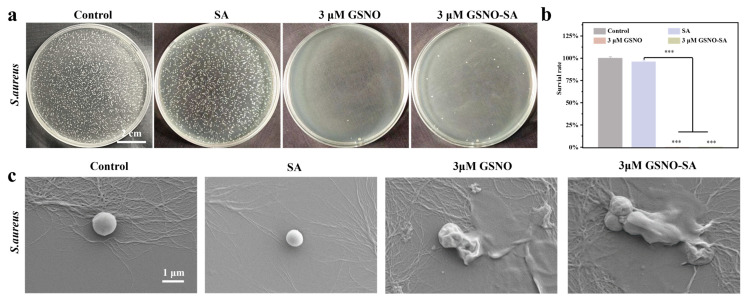
(**a**) Representative images of wound tissue bacteria in each treatment group on day 3. (**b**) Bacterial survival rate. (**c**) SEM images of and *S. aureus* after co-culture with different treatment groups for 12 h. (*** *p* < 0.001, *n* = 3).

Three days post-surgery, wound tissues from rats in each group were collected to evaluate the infection status. As shown in Figure 9a, dense bacterial colonies were observed in the control and SA groups, indicating severe infection in these groups. In contrast, the 3 μM GSNO and the 3 μM GSNO-SA groups effectively cleared the bacteria. The antibacterial effect of the 3 μM GSNO-SA group was comparable to that of the 3 μM GSNO group, suggesting that adding GSNO to SA did not weaken the antibacterial activity of GSNO. The quantification of bacterial survival, shown in Figure 9b, further confirmed this. The bacterial survival rate in the SA group was nearly 100%, indicating that the hydrogel itself has no significant antimicrobial activity. In contrast, the bacterial survival rate in the 3 μM GSNO group was almost zero, and the survival rate in the 3 μM GSNO-SA group was also very low, at 0.25 ± 0.04%. Statistical analysis confirmed that the antibacterial effects of both the 3 μM GSNO and 3 μM GSNO-SA groups were significant (*** *p* < 0.001).

GSNO and GSNO-SA demonstrated significant antimicrobial activity. To further examine this, SEM was employed to observe the bacterial morphology following treatment with the experimental groups. As depicted in Figure 9c, treatment with the control and SA groups resulted in *S. aureus* maintaining its intact morphology, exhibiting a smooth surface with no visible damage, indicating that these treatments did not cause direct structural disruption. In contrast, in the 3 μM GSNO and 3 μM GSNO-SA groups, marked changes in bacterial morphology were observed. *S. aureus* exhibited membrane disruption and dissolution, with some bacterial cells aggregating and undergoing lysis. These findings suggest that NO released from GSNO may exert potent bactericidal effects by disrupting membrane integrity, causing osmotic imbalance, or directly inducing bacterial lysis.

### 2.8. Immunohistochemical and Immunofluorescent Analysis

**Figure 10:**
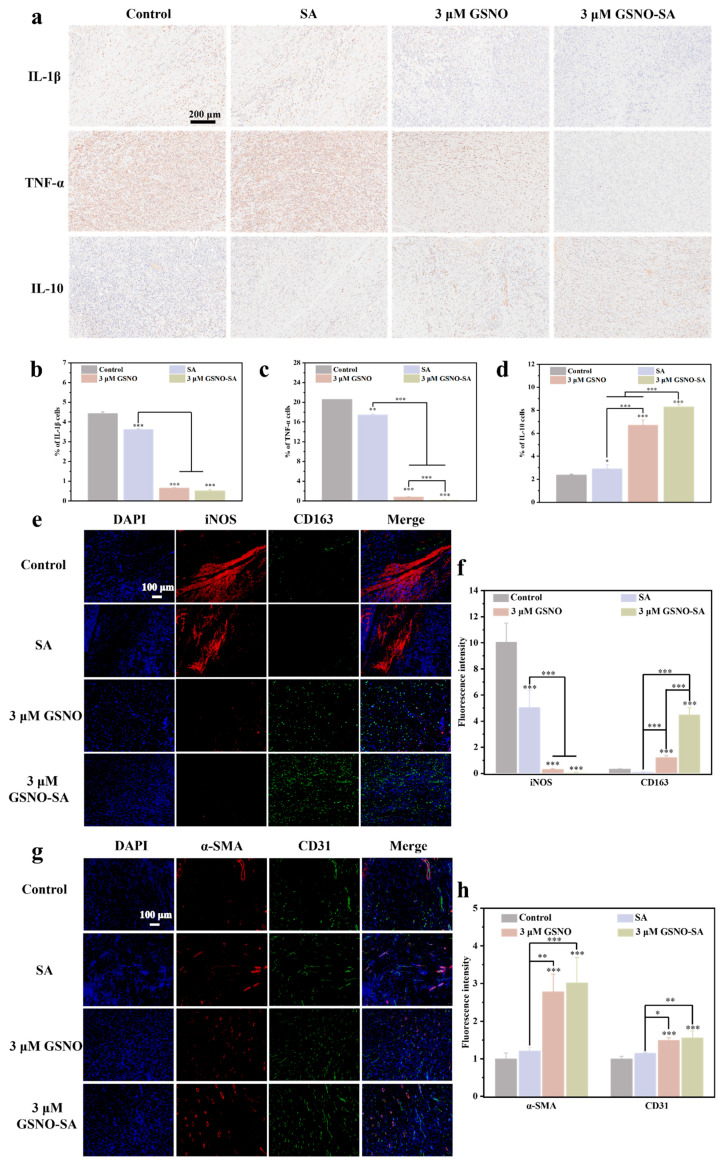
(**a**) Immunohistochemical staining images of IL-1β, TNF-α, and IL-10 in wounds from each group on day 3. (**b**) Quantitative statistical graphs: IL-1β, (**c**) TNF-α, (**d**) IL-10. (**e**) Representative immunofluorescence staining images of DAPI (blue), iNOS (red) and CD163 (green) in wound tissues of different groups on day 3. (**f**) Quantitative analysis of iNOS and CD163. (**g**) Representative immunofluorescence staining images of DAPI (blue), α-SMA (red) and CD31 (green) in wound tissues of different groups on day 7. (**h**) Quantitative analysis of α-SMA and CD31. (* *p* < 0.05, ** *p* < 0.01, *** *p* < 0.001, *n* = 3).

Local inflammation plays a key role in wound healing. Inflammation, on the one hand, controls the process of tissue recovery. On the other hand, the phenotype of inflammatory cells controls the degree of angiogenesis. As shown in Figure 10a–d, the expression of IL-1β and TNF-α was most prominent in the control and SA groups, while IL-10 expression remained relatively low. This indicates a stronger inflammatory response in these groups, which may lead to a prolonged inflammatory state and impair wound healing efficiency, primarily due to bacterial infection at the wound site. In contrast, both the 3 μM GSNO and 3 μM GSNO-SA groups significantly reduced the expression of IL-1β and TNF-α. Additionally, the 3 μM GSNO-SA group notably enhanced IL-10 expression, increasing it by 3.49-fold compared to the control group, suggesting its role in inflammation regulation. This effect is likely attributed to the SA hydrogel’s ability to absorb exudate while releasing NO, which modulates the inflammatory response, decreases pro-inflammatory factor production, and ultimately accelerates wound healing.

Figure 10e,f presents the expression levels of iNOS and CD163 in the different experimental groups. In the control group, iNOS expression was most pronounced, while CD163 expression was nearly undetectable, indicating a severe inflammatory response at the wound site. In the SA group, iNOS expression decreased by 49.9% compared to the control, while CD163 expression showed only a slight reduction. This suggests that, while the hydrogel effectively absorbed exudates, its anti-inflammatory effect was limited. In the 3 μM GSNO group, iNOS expression decreased significantly, and CD163 expression was notably enhanced (*** *p* < 0.001), indicating that NO promoted the transition of macrophages from the M1 to M2 phenotype, thereby accelerating the resolution of inflammation. The 3 μM GSNO-SA group exhibited the lowest iNOS expression and the highest CD163 expression, suggesting that the GSNO-SA hydrogel was most effective in reducing inflammation and promoting tissue repair. This can be attributed to the ability of NO to suppress the NF-κB signaling pathway, thereby reducing the release of pro-inflammatory cytokines, and to induce the polarization of M1 macrophages into the M2 phenotype through the VASP signaling pathway [33,34].

α-SMA and CD31 are key markers of angiogenesis. Immunofluorescence staining of α-SMA and CD31 was performed to evaluate the presence of newly formed blood vessels in granulation tissue. Figure 10g,h presents the immunofluorescence staining results for α-SMA and CD31 on day 7 across different experimental groups. The expression levels of α-SMA in the groups were 100 ± 15%, 121 ± 9%, 278 ± 47%, and 302 ± 66%, respectively, while the expression levels of CD31 were 100 ± 6%, 115 ± 2%, 150 ± 7%, and 156 ± 15%. In the 3 μM GSNO group, the expression of both α-SMA and CD31 was significantly increased (*** *p* < 0.001), with the highest expression levels observed in the 3 μM GSNO-SA group. These findings indicate that the 3 μM GSNO-SA group exhibited the most pronounced angiogenesis during the early phase of wound healing. This can be attributed to NO, which enhanced the stability of hypoxia-inducible factor-1α (HIF-1α) by inhibiting prolyl hydroxylase (PHD). The elevated expression of HIF-1α subsequently promoted the secretion of VEGF, which was regarded as one of the most critical growth factors in the wound healing process. VEGF facilitated the proliferation and differentiation of endothelial cells and induced the formation of new blood vessels [35,36].

## 3. Conclusions

This study successfully developed an innovative GSNO-SA hydrogel to address the significant challenges in chronic wound healing, particularly those associated with infection, inflammation, and delayed healing processes. The hydrogel demonstrated prolonged NO release for up to 48 h, enhancing exudate management, exhibiting strong antibacterial properties, and promoting angiogenesis and anti-inflammatory effects. In vitro results confirmed the hydrogel’s ability to inhibit bacterial growth, regulate inflammation, and support endothelial cell migration, which are crucial for wound healing. Additionally, in vivo experiments showed that the GSNO-SA hydrogel significantly accelerated wound healing, achieving nearly complete closure (99.2 ± 0.1%) within 14 days, outperforming conventional treatments. Histological and immunohistochemical assessments further validated the hydrogel’s therapeutic efficacy, highlighting its capacity to regulate inflammation and promote vascular regeneration. This research provides a novel material platform that combines NO-based therapeutic strategies with hydrogel exudate management for chronic wound care, offering substantial potential for advancing clinical treatments and improving patient outcomes in the management of chronic, infected wounds.

## 4. Materials and Methods

### 4.1. Materials

Starting materials, reagents, and solvents were purchased from commercial sources, were of analytical grade, and were used as received without further purification. Reduced glutathione (GSH) was obtained from Shanghai Titan Technology Co., Ltd. (Shanghai, China). Sodium alginate (SA) was sourced from McLean Biochemical Technology Co., Ltd. (Shanghai, China). Calcium chloride (CaCl_2_) was acquired from Sigma-Aldrich Trading Co., Ltd. (Shanghai, China). Sodium nitrite (NaNO_2_) was purchased from Meryer Chemical Technology Co., Ltd. (Shanghai, China). Griess reagent was supplied by Beyotime Biotechnology Co., Ltd. (Shanghai, China). Dulbecco’s Modified Eagle Medium (DMEM) and penicillin–streptomycin solution (PS) were purchased from Gibco BRL (Beijing, China). The Cell Counting Kit-8 assay (CCK-8) was purchased from Dojindo Molecular Technologies Inc. (Shanghai, China). The Live-Dead Cell Staining Kit (AO-EB) was purchased from Beijing Solarbio Science & Technology Co., Ltd. (Beijing, China).

### 4.2. Synthesis and Characterization of GSNO

The preparation of GSNO was carried out based on the method described in the literature [37]. A total of 4.8 g of GSH was weighed and dissolved in 25 mL of 0.63 mol/L HCl solution. NaNO_2_ was then added in a 1:1 molar ratio, and the mixture was stirred for 40 min under dark and ice-cold conditions. Subsequently, 25 mL of cold acetone was added, and the mixture was vacuum-filtered. The residue was washed once with 10 mL of an 80:20 (*v*/*v*) acetone–water solution, followed by two washes with 10 mL of acetone, and then three washes with 10 mL of ether. The resulting pink solid was dried in a vacuum oven at 27 °C for 12 h.

Fourier transform infrared spectroscopy (FT-IR, PerkinElmer, Waltham, MA, USA), ultraviolet-visible spectrophotometry (UV-2550, Shimadzu Corporation, Suzhou, China), and proton nuclear magnetic resonance (^1^H-NMR) were employed to analyze GSH and GSNO.

### 4.3. Detection of NO Release of GSNO

Standard solutions of 0, 10, 250, 500, 1000, and 1500 nM were prepared, each with a volume of 50 μL, and three parallel samples were prepared for each group. Subsequently, 50 μL of Griess Reagent I was added, followed by the addition of 50 μL of Griess Reagent II. Absorbance (OD) values were measured at 540 nm using a multifunctional microplate reader. A standard curve was constructed by correlating concentration with absorbance, as follows: A = 0.00106C + 0.03872, R^2^ = 0.9999, where A is the absorption value, and C is the NO concentration with a R^2^ value of 0.9999.

GSNO powder (15.12, 10.08, 5.04, 1.01 and 0.34 μg) was dissolved in 10 mL of phosphate buffer brine (PBS, pH = 7.4) to prepare solutions with concentrations of 4.5, 3.0, 1.5, 0.3, and 0.1 μM, and three parallel samples were prepared for each group. To simulate the physiological fluid environment, a beaker filled with water at 37 °C was prepared, and a magnetic stir bar was added to induce circulation. The solutions were placed in a centrifuge tube, sealed with parafilm, and subsequently immersed in the stirred water bath. At 1, 3, 6, and 12 h, 50 μL of each solution was collected, diluted 10-fold, and transferred to a 96-well plate. To each well, 50 μL of Reagent I and 50 μL of Reagent II were added, and the reaction was incubated in the dark for 30 min. Afterward, absorbance was measured at 540 nm using a microplate reader. NO release at different GSNO concentrations was calculated from the standard curve.

### 4.4. In Vitro Antibacterial and Cytocompatibility of GSNO

Experimental details of in vitro antibacterial and cytocompatibility of GSNO are provided in the Supporting Information, and three parallel samples were prepared for each group.

### 4.5. Synthesis and Characterization of GSNO-SA Hydrogel

To prepare the GSNO-SA hydrogel, a 2% (*w*/*v*) SA solution was first prepared. GSNO was then added to achieve final concentrations of 3.0 μM, 1.5 μM, and 0.3 μM, and the mixture was stirred under light-protected conditions at 25 °C for 10 min. The resulting solution was frozen at –20 °C for 8 h. It was subsequently transferred to a 4 °C refrigerator, and 0.2 mL of 2% (*w*/*v*) calcium chloride (CaCl_2_) solution was added dropwise onto the surface of the frozen solution within 2 min. The mixture was then left undisturbed for 12 h.

The morphology of the microspheres and hydrogels was observed using a scanning electron microscope (SEM, ULTRA55, Carl Zeiss, Baden-Württemberg, Germany). The elemental distribution of the hydrogels was subsequently recorded using an energy-dispersive X-ray spectroscopy system (EDS, K-Alpha+, Thermo Scientific, Waltham, WA, USA).

### 4.6. Water Absorption and Swelling

For a water absorption test, a 0.01% (*w*/*v*) crystal violet solution (7.85 mL) was prepared using PBS and poured into a dish (Φ 10 cm), ensuring the solution covered the bottom of the dish. The GSNO-SA hydrogels were then placed into the dish, and photographs were taken to record the hydrogel’s absorption of the solution.

For a swelling test, the GSNO-SA hydrogels were immersed in 37 °C PBS, respectively, and three parallel samples were prepared for each group. The initial mass of the samples was denoted as W_0_. The vial was sealed at 37 °C for 24 h, and the samples were taken out followed by the removal of excess water from the hydrogel surface, whose mass was denoted as W_X_. The equilibrium swelling ratio (Q_X_) was calculated using the following Equation (1):

(1) $$\;Q_{X}(\%)=\frac{W_{X}-W_{0}}{W_{0}}\times \;100\%$$

### 4.7. Detection of NO Release of GSNO-SA Hydrogel

The amount of GSNO added during the preparation of the GSNO-SA hydrogel was determined based on the total volume of the experimental system, ensuring final concentrations of GSNO in the hydrogel of 3.0, 1.5, and 0.3 μM, and three parallel samples were prepared for each group. To simulate the physiological fluid environment, a beaker filled with water at 37 °C was prepared, and a magnetic stir bar was added to induce circulation. The hydrogel sample was placed in a centrifuge tube containing 10 mL of PBS solution, sealed with parafilm, and subsequently immersed in the stirred water bath. At predefined time points, 50 μL of supernatant was collected, and the NO release was quantified using the Griess reagent and the standard curve.

### 4.8. Mechanical Properties of GSNO-SA Hydrogel

The experimental details for the compression and tensile tests of GSNO-SA hydrogels at various concentrations are provided in the Supplementary Information, and three parallel samples were prepared for each group.

### 4.9. In Vitro Antibacterial of GSNO-SA Hydrogel

The antimicrobial properties of GSNO-SA hydrogel at varying concentrations were assessed using plate coating, inhibition zones, and inhibition curves. Experimental details are provided in the Supporting Information, and three parallel samples were prepared for each group.

### 4.10. In Vitro Cytocompatibility of GSNO-SA Hydrogel

Experimental details of the in vitro cytocompatibility of the GSNO-SA hydrogel are provided in the Supporting Information, and three parallel samples were prepared for each group.

### 4.11. Cell Migration

The GSNO-SA hydrogel was co-cultured with HUVECs to evaluate its cell compatibility. Experimental details are provided in the Supporting Information, and three parallel samples were prepared for each group.

### 4.12. PCR Analysis of Inflammatory Factors and Angiogenesis-Related Factors

The objective of this experiment was to assess the effects of a GSNO-SA hydrogel on angiogenesis-related factors VEGF, TGF-β, and CD31 in HUVECs and on the expression of inflammation-related factors TNF-α, iNOS, and CD206 in RAW264.7 mouse macrophages, using the indirect contact method with Transwell chambers. Experimental details are provided in the Supporting Information, and three parallel samples were prepared for each group.

### 4.13. In Vivo Wound Healing

Sixteen male SD rats (8-week-old) were acclimated to the laboratory conditions for 3 days. The average weight of all rats was 246 ± 23 g. Animal experiments, conducted in accordance with the National Research Council’s Guide for the Care and Use of Laboratory Animals, were reviewed and approved by the Animal Ethics Committee at the Animal Experimental Center of Guangdong Pharmaceutical University (Approval No. 20240301-002), China. To simulate ischemia/reperfusion injury, symmetric strong rare-earth magnets (Φ 10 mm, 0.5–0.6 T) were used to clamp the skin marked on the back of rats. After 12 h of clamping, the magnets were removed, and the procedure was repeated for five cycles, with 12 h intervals between each cycle, resulting in the formation of four pressure ulcers. Wounds were created by excising full-thickness skin (10 mm in diameter) using sterile surgical instruments, with four wounds being prepared on each rat. A 100 µL suspension of *S. aureus* (1 × 10^7^ CFU/mL) was applied to each wound and was evenly distributed. Treatment began on day 0, with wounds being treated according to the assigned group (Control, SA hydrogel, 3 μM GSNO, or 3 μM GSNO-SA). Breathable tape was used to secure the dressings, which were changed every two days. Wound progression was monitored on days 0, 3, 7, 10, and 14 through photography, and tissue samples were collected for further analysis. On days 0, 3, 7, 10, and 14, rats were anesthetized via intraperitoneal injection of 1% pentobarbital sodium (50 mg/kg), and wound images were captured. Wound areas were measured, and the wound healing rate was calculated using the following Formula (2):

(2) $$\;\mathrm{Wound}\;\mathrm{healing}\;\mathrm{rate}\;(\%)=\frac{S_{o}-S_{t}}{S_{o}}\;\times \;100$$

S_0_ denotes the initial wound area, while S_t_ represents the wound area on day t.

### 4.14. In Vivo Antibacterial

On day 3, skin tissue was collected from the wound, and an appropriate amount of tissue was placed into a 5 mL sterile centrifuge tube, to which sterile PBS was added. The tissue suspension in each group was then diluted 10,000-fold, and 100 μL of the diluted solution was plated onto agar. Three parallel samples were prepared for each group. After plating, the agar plates were incubated at 37 °C with 5% CO_2_ for 12 h. Following incubation, images of the agar plates were captured and recorded for further analysis.

To examine bacterial morphology, bacteria collected from the samples were centrifuged (6000 rpm, 15 min), and the bacterial pellet was incubated overnight with 1.5% glutaraldehyde. The bacterial samples were subsequently dehydrated using a series of ethanol solutions (30%, 50%, 70%, 80%, 90%, 100%). To ensure the observation of individual bacteria during imaging, the dehydrated bacterial suspension was diluted 10,000-fold. A small volume of the diluted suspension was then placed onto silicon wafers and air-dried in a fume hood. The morphology of the bacteria from each treatment group was observed using scanning electron microscopy.

### 4.15. Histologic and Immunohistochemistry

The inflammatory response and tissue regeneration processes were investigated. On days 3, 7, 10, and 14, skin tissue samples were collected from the wound and fixed in 4% paraformaldehyde, and three parallel samples were prepared for each group. After paraffin embedding, the specimens were sectioned into 5 μm thick slices for histological analysis. The sections were stained with H&E and Masson’s trichrome stain and examined using high-resolution optical microscopy. On day 3, immunohistochemical staining for IL-1β, TNF-α, and IL-10 was performed, followed by immunofluorescence labeling for iNOS and CD163. On day 7, immunofluorescence labeling for CD31 and α-SMA was conducted.

### 4.16. Statistical Analysis

All data were analyzed using Graphpad prism 7.0 software (one-way ANOVA), and the results were expressed as the mean ± standard deviation (SD), and the results of significant differences were as follows: * *p* < 0.05, ** *p* < 0.01, *** *p* < 0.001, *n* = 3.

## Data Availability

The original contributions presented in this study are included in the article/Supplementary Materials. Further inquiries can be directed to the corresponding authors.

## Supplementary Materials

The following supporting information can be downloaded at: https://www.mdpi.com/article/10.3390/gels11050354/s1, Supporting Information is available from the Wiley Online Library or from the author. Table S1. Real-time release rate of GSNO-SA hydrogel. Table S2. Sequences of vascularization primer. Table S3. Sequences of inflammatory primer. Figure S1. Standard curve of NO. Figure S2. Real-time release curve of GSNO-SA hydrogel. Figure S3. (a) Compression strength bar chart of GSNO-SA hydrogel. (b) Tensile strength bar chart of GSNO-SA hydrogel. Figure S4. (a) Korsmeyer–Peppas model fitting curve of 3 μM of GSNO-SA hydrogel. (b) Korsmeyer–Peppas model fiting curve of 1.5 μM of GSNO-SA hydrogel. (c) Korsmeyer–Peppas model fiting curve of 0.3 μM of GSNO-SA hydrogel. Figure S5. (a) Live/dead staining of Hacat cells after treatment with GSNO-SA hydrogel. (b) Viability of Hacat cells after treatment with GSNO-SA hydrogel. (* *p* < 0.05, ** *p* < 0.01, *** *p* < 0.001, *n* = 3). Figure S6. (a) Live/dead staining of L929 cells after treatment with GSNO-SA hydrogel. (b) Viability of L929 cells after treatment with GSNO-SA hydrogel. (* *p* < 0.05, *** *p* < 0.001, *n* = 3).
