# Supplementary material for: Development of an Asymmetric Alginate Hydrogel Loaded with S-Nitrosoglutathione and Its Application in Chronic Wound Healing
Source: Gels. 2025 May 12;11(5):354. doi: 10.3390/gels11050354 (PMC12111571; doi:10.3390/gels11050354)
Supplement: Supplementary file 1 [file gels-11-00354-s001.zip › gels-3618937-supplementary.pdf]

1. *In vitro* antibacterial and cytocompatibility of GSNO

Escherichia coli (*E. coli*) and *Staphylococcus aureus* (*S. aureus*) were selected as the bacterial strains, and the antimicrobial effects of different GSNO concentrations were evaluated using the plate spread method. GSNO powder of varying amounts was added to 15 mL centrifuge tubes, followed by 5 mL of broth. The mixture was gently shaken and vortexed to facilitate complete dissolution. *E. coli* and *S. aureus* bacterial suspensions were then introduced, and the volume was adjusted to 10 mL, ensuring a final bacterial concentration of 10^4 CFU/mL, with GSNO concentrations set at 4.5, 3.0, 1.5, 0.3, and 0.1 μM. The suspensions were incubated at 37°C for 12 hours, after which they were diluted 10,000-fold with broth. Fifty microliters of the diluted suspension was applied to the surface of agar plates and spread evenly using a spreading bead. The plates were incubated at 37°C in a CO_2 incubator for 24 hours, and the results were recorded by photographing the plates.

To assess the effect of different GSNO concentrations on cell proliferation, a CCK-8 assay was conducted. The procedure was as follows: GSNO solutions at concentrations of 4.5, 3.0, 1.5, 0.3, and 0.1 μmol/L were prepared using cell culture medium. Resuspended HUVECs were seeded at a density of 10_4 cells per well in a 12-well plate and co-cultured with the respective GSNO concentrations for 1, 3, and 5 days. At these time points, a CCK-8 solution was prepared at a 1:10 ratio (CCK-8 reagent to culture medium), with 0.3 mL added to each well. After 2 hours of incubation in the dark, absorbance was measured at 450 nm using a microplate reader. The experimental group without GSNO served as the control, while the group without cells acted as the blank. Cell viability was calculated using the following formula:

$$\text{Cell Viability}=\frac{OD_t\text{-}OD_0}{OD_c\text{-}OD_0}\times 100\%$$

where OD_0 represents the absorbance of the blank group, OD_c represents the absorbance of the control group, and OD_t represents the absorbance of the test group.

Cell morphology was observed using acridine orange/ethidium bromide (AO/EB) dual fluorescence staining. At specific time points, the culture medium was removed and washed with PBS buffer. Subsequently, 0.1 mL of AO/EB solution (AO:EB:PBS

= 1:1:1000) was added, and the cells were incubated in the dark for 30 minutes. After incubation, the staining solution was aspirated, and red and green fluorescence of the cells was observed using a fluorescence microscope. Images were captured from different locations.

2. Mechanical properties of GSNO-SA hydrogel

The mechanical properties of the hydrogel, including compressive and tensile behaviors, were evaluated using a universal testing machine.

Compressive testing: The samples were shaped into cylinders (Φ 10 mm× 10 mm). During testing, a compressive load was applied at a rate of 1 mm/min, and the compressive strength was defined as the stress value when the compressive strain reached 80%.

Tensile testing: The sample preparation method for tensile testing was the same as compressive testing, except that a strip-shaped mold (80 mm × 15 mm × 1 mm) was used. Each group included at least three parallel samples. During testing, a tensile load was applied at a rate of 1 mm/min, and the tensile strength was defined as the maximum stress value the sample could withstand.

3. In vitro antibacterial of GSNO-SA hydrogel

The antibacterial activity of the GSNO-SA hydrogel was evaluated using the following three methods: inhibition zones, antibacterial curves, and bacterial plate counting.

Bacterial Plate Counting Method: Different concentrations of the GSNO-SA hydrogel were added to 15 mL centrifuge tubes containing 5 mL of broth medium. Suitable volumes of *E. coli* and *S. aureus* suspensions were introduced, and the final volume was adjusted to 10 mL to achieve a bacterial concentration of 10^4 CFU/mL, with final GSNO concentrations of 3.0, 1.5, and 0.3 μM. The suspensions were incubated at 37℃ for 12 hours, followed by a 10,000-fold dilution in broth. A 50 μL aliquot of the diluted suspension was plated onto agar plates and evenly spread using an inoculation loop. The plates were incubated at 37 ℃ in a CO_2 incubator for 24 hours, and the results were documented by photographing the plates.

Inhibition Zone Method: Fifty μL aliquots of *E. coli* and *S. aureus* suspensions (10^4 CFU/mL) were evenly spread on the surface of agar plates. Using a punch, three holes were made in the agar to match the size of the hydrogel samples. The GSNO-SA hydrogel samples were placed into the wells. The plates were sealed with adhesive film to prevent contamination and evaporation, and they were incubated at 37 °C in a CO_2 incubator for 12 hours. The presence of inhibition zones around the hydrogel was observed, and the results were recorded using a camera.

Antibacterial Curve Method: Different concentrations of GSNO-SA hydrogel were added to 15 mL centrifuge tubes containing 5 mL of broth medium. Appropriate volumes of *E. coli* and *S. aureus* suspensions were added, and the final volume was adjusted to 10 mL, resulting in a bacterial concentration of 10^4 CFU/mL and final GSNO concentrations of 3.0, 1.5, and 0.3 μM. The samples were incubated in a 37 °C shaking incubator at predetermined time points. After incubation, 100 μL of bacterial suspension was transferred to a 96-well plate, and the absorbance at 600 nm was measured using a multifunctional microplate reader to assess bacterial growth.

4. In vitro cytocompatibility of GSNO-SA hydrogel

HUVEC cell suspension (1.5 mL, 10^4 cells/well) was seeded in a 12-well culture plate and incubated at 37 °C with 5% CO_2 for 12 hours. A Transwell chamber with a pore size of 0.4 μm was then placed into each well, followed by the addition of different concentrations of GSNO-SA hydrogel and 0.5 mL of cell culture medium.. On days 1, 3, and 5, 0.3 mL of CCK-8 solution was added to each well, followed by 2-hour incubation in the dark. Absorbance at 450 nm was measured using a microplate reader. The group without GSNO served as the control, and the group without cells was used as the blank. Cell viability was calculated using the following formula:

$$\text{Cell Viability}=\frac{OD_t\text{-}OD_0}{OD_c\text{-}OD_0}\times 100\%$$

where OD_0 represents the absorbance of the blank group, OD_c represents the absorbance of the control group, and OD_t represents the absorbance of the test group.

Cell morphology was observed using acridine orange/ethidium bromide (AO/EB) dual fluorescence staining. At specific time points, the culture medium was removed

and washed with PBS buffer. Subsequently, 0.1 mL of AO/EB solution (AO:EB:PBS = 1:1:1000) was added, and the cells were incubated in the dark for 30 minutes. After incubation, the staining solution was aspirated, and red and green fluorescence of the cells was observed using a fluorescence microscope. Images were captured from different locations.

The in vitro compatibility experiments involving human keratinocytes cells (Hacat) and NCTC clone 929 (L929) were conducted following the same procedure as described above.

5. Cell migration

To examine the effect of hydrogels on cell migration, a cell scratch assay was performed. HUVECs were seeded in a 12-well plate at a density of 6×10^4 cells per well and cultured with 1 mL of medium containing 1% fetal bovine serum and 1% penicillin-streptomycin for 24 hours. A scratch was created using a pipette tip, and the wells were washed twice with PBS. Subsequently, 1 mL of medium without fetal bovine serum was added, and Transwell chambers were placed in the wells with different concentrations of GSNO-SA hydrogel. At 24 and 48 hours, the Transwell chambers were removed, the medium was discarded, and 1 mL of PBS buffer was added. Cell migration was then observed under a microscope. The cell scratch closure rate was calculated using the following formula:

$$\text{Cell scratch healing rate (\%)}=\frac{S_0-S_t}{S_0}\times100$$

where S_0 denotes the initial scratch area, and S_t represents the scratch area after intervention.

6. PCR Analysis

An amount of 1.5 mL of HUVEC cell suspension (at a density of 2×10^4 cells/well) was added to each well of a 12-well culture plate. A Transwell chamber was then placed in each well, and varying concentrations of GSNO-SA hydrogel were introduced into the chamber. Subsequently, 0.5 mL of cell culture medium was added to each Transwell chamber. After 72 hours, cells were harvested, and total RNA was

extracted using Trizol reagent. mRNA expression was quantified using real-time PCR. Primer sequences are provided in Table S2.

An amount of 2 mL of RAW264.7 cell suspension (10^5 cells/well) was added to each well of a 12-well plate. After incubating the RAW264.7 cells for 12 hours, the original culture medium was removed, and 1 μg/mL of lipopolysaccharide (LPS) was introduced into the wells. After another 12 hours, the LPS-containing medium was removed, and 1.5 mL of fresh culture medium was added to each well. A Transwell insert was placed in each well, and 0.5 mL of GSNO-SA hydrogel was introduced into each insert. Additionally, 0.5 mL of culture medium was added to each Transwell insert. After 72 hours, the cells were collected, and total RNA was extracted using Trizol reagent. mRNA expression was quantified by real-time PCR. The primer sequences are provided in Table S3.

Table S1. Real-time release rate of GSNO-SA hydrogel.

Time	3 μM GSNO-SA	1.5 μM GSNO-SA	0.3 μM GSNO-SA
0-1 h	269.53 ± 9.17	107.99 ± 7.26	22.09 ± 1.47
1-3 h	65.06 ± 7.82	37.18 ± 3.48	14.58 ± 2.92
3-6 h	14.00 ± 3.06	26.50 ± 1.47	11.97 ± 0.19
6-12 h	23.08 ± 5.02	15.87 ± 1.12	3.74 ± 0.56
12-24 h	8.68 ± 0.86	5.61 ± 0.21	1.39 ± 0.37
24-36 h	10.76 ± 0.49	2.27 ± 0.73	0.45 ± 0.28
36-48 h	1.79 ± 1.56	1.23 ± 0.44	0.21 ± 0.30
48-72 h	0.66 ± 0.52	0.34 ± 0.21	0 ± 0.30

Table S2. Sequences of vascularization primer.

mRNA	Forward primer	Reverse primer
VEGF	CGGTATAAGTCCTGGAGCGT	TTTAACTCAAGCTGCCTCGC
CD31	CCAAGGTGGGATCGTGAGG	TCGGAAGGATAAAACGCGGTC
TGF-β	TACAGCAACAATTCCTGGCG	GTGAACCCGTTGATGTCCAC
GAPDH	GGAGCGAGATCCCTCCAAAAT	GGCTGTTGTCATACTTCTCATGG

Table S3. Sequences of inflammatory primer.

mRNA	Forward primer	Reverse primer
TNF-α	AACCTCCTCTCTGCCATCAA	GGAAGACCCCTCCCAGATAG
iNOS	CAACAGGGAGAAAGCGCAAA	TGATGGACCCCAAGCAAGAC
CD206	AGCTTCATCTTCGGGCCTTTG	GGTGACCACTCCTGCTGCTTTAG
GAPDH	AGGAGCGAGACCCCACTAACA	AGGGGGGCTAAGCAGTTGGT

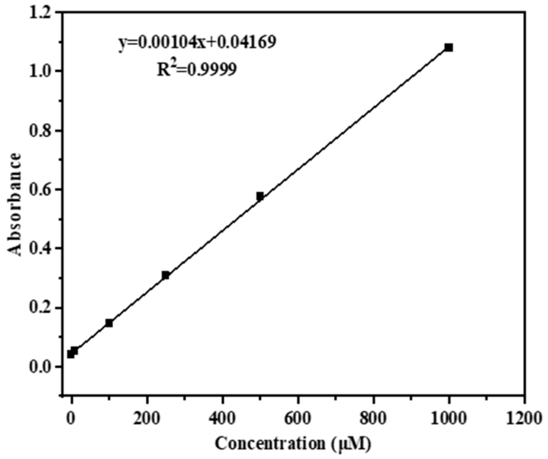

Figure S1 . Standard curve of NO.

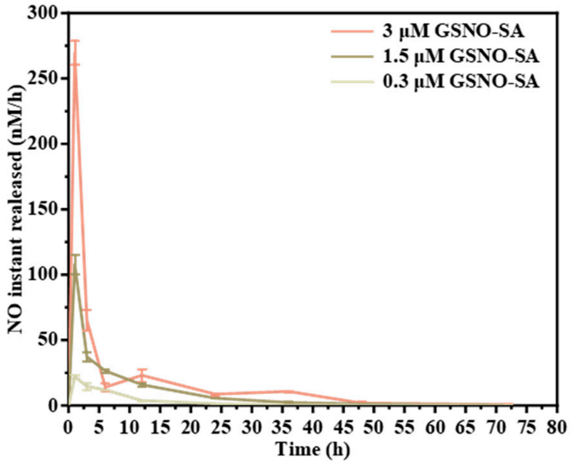

Figure S2 . Real-time release curve of GSNO-SA hydrogel.

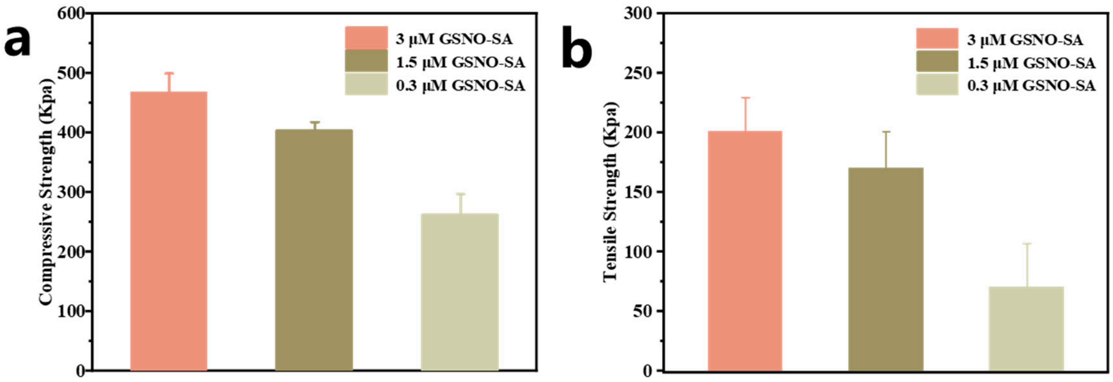

Figure S3 . (a) Compression strength bar chart of GSNO-SA hydrogel. (b) Tensile strength bar chart of GSNO-SA hydrogel.

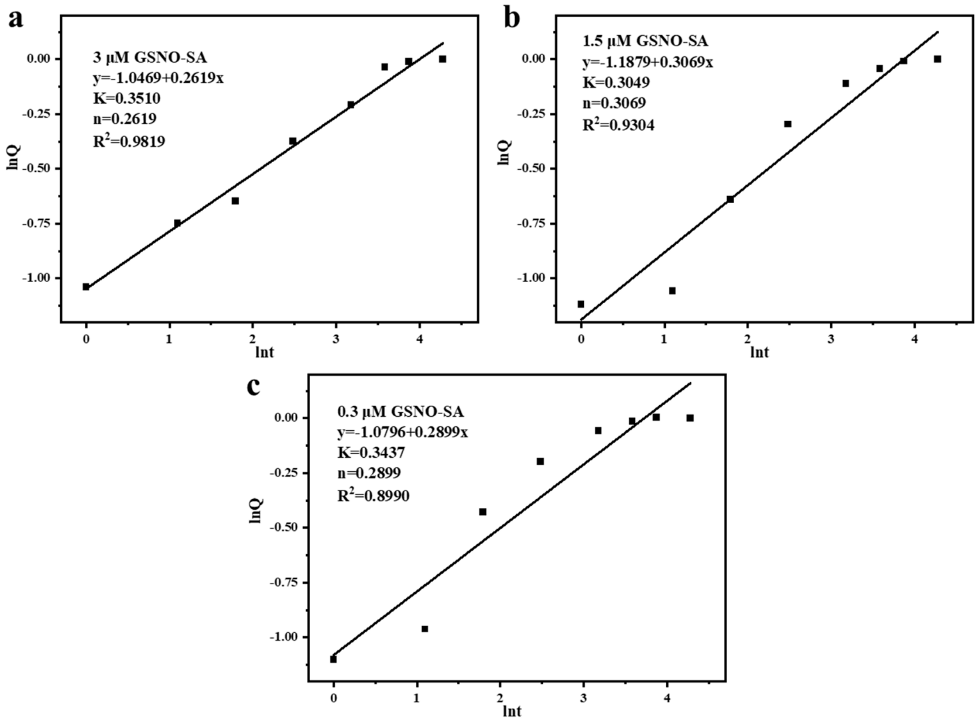

In these pictures, Q represents the ratio of M_t to M_∞, where Mt is the cumulative amount of GSNO released at time t, and M_∞ is the total amount of GSNO released. The unit of t is hours.

Figure S4 . (a) Korsmeyer–Peppas model fitting curve of 3 μM of GSNO-SA hydrogel. (b) Korsmeyer–Peppas model fiting curve of 1.5 μM of GSNO-SA hydrogel. (c) Korsmeyer–Peppas model fiting curve of 0.3 μM of GSNO-SA hydrogel.

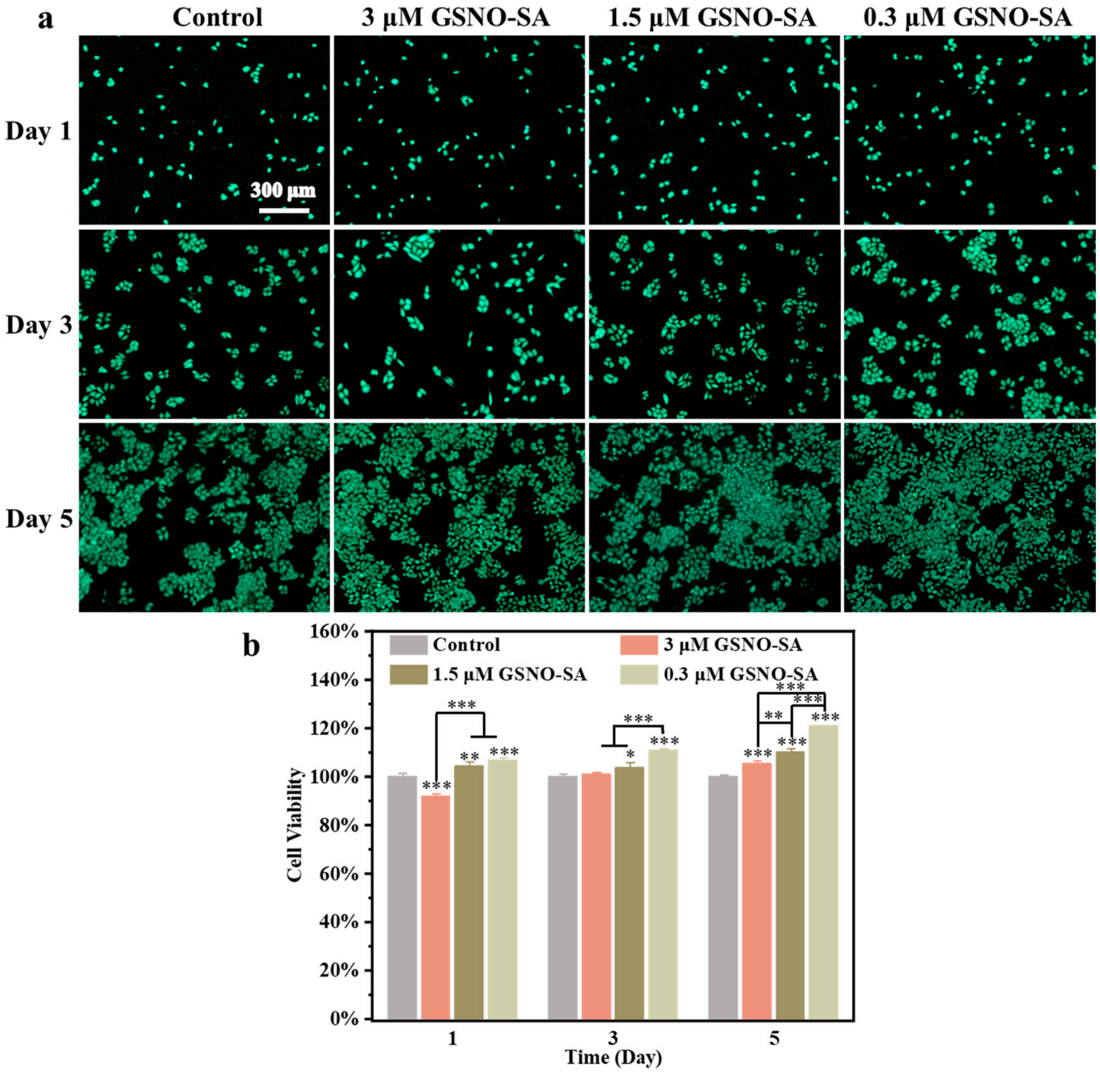

Figure S5 . (a) Live/dead staining of Hacat cells after treatment with GSNO-SA hydrogel. (b) Viability of Hacat cells after treatment with GSNO-SA hydrogel. (*$p < 0.05$, **$p < 0.01$, ***$p < 0.001$, n = 3).

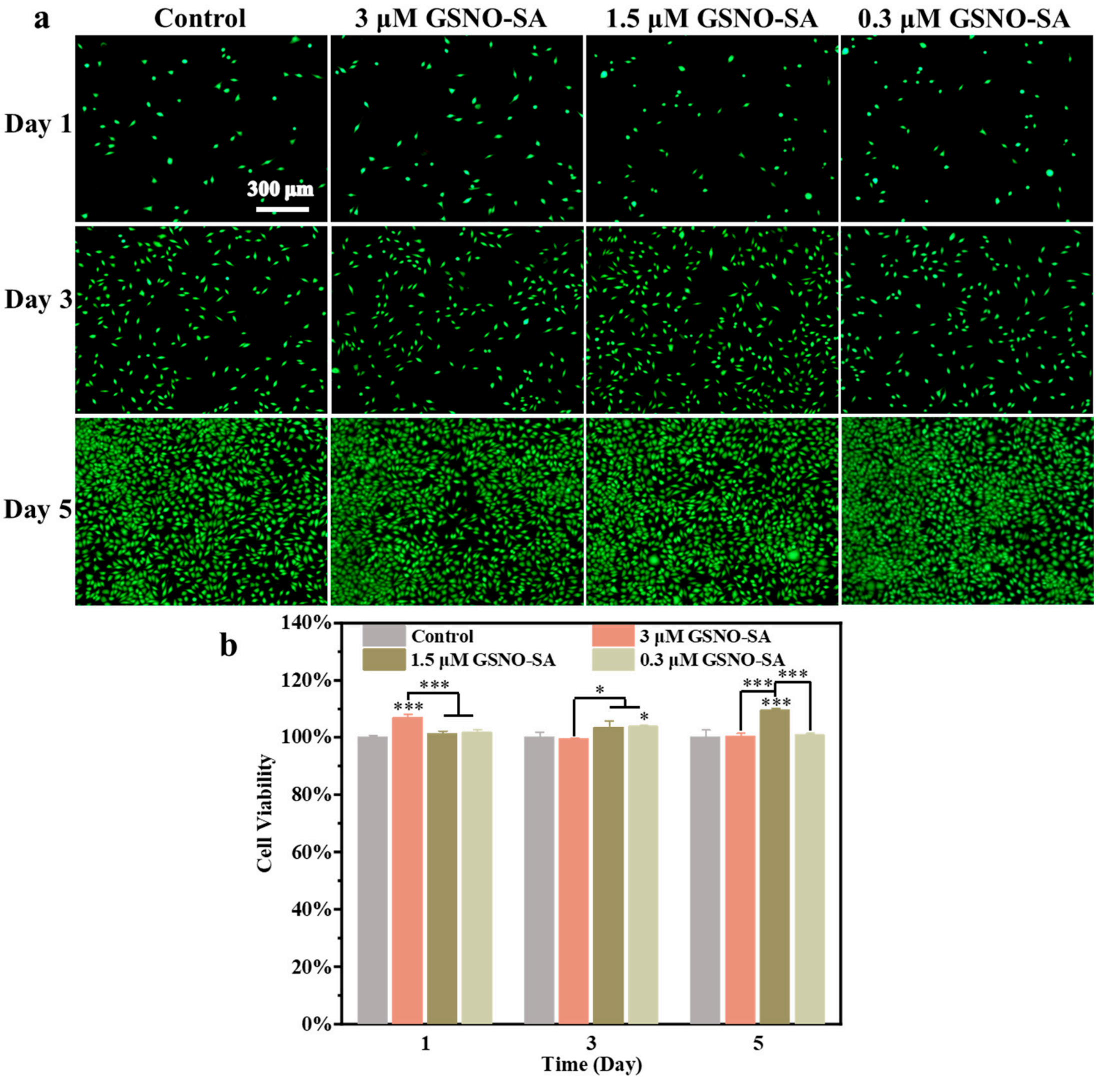

Figure S6 . (a) Live/dead staining of L929 cells after treatment with GSNO-SA hydrogel. (b) Viability of L929 cells after treatment with GSNO-SA hydrogel. (*$p < 0.05$, ***$p < 0.001$, $n = 3$).
